# Dolabelladienetriol, a Compound from *Dictyota pfaffii* Algae, Inhibits the Infection by *Leishmania amazonensis*

**DOI:** 10.1371/journal.pntd.0001787

**Published:** 2012-09-06

**Authors:** Deivid Costa Soares, Teresa C. Calegari-Silva, Ulisses G. Lopes, Valéria L. Teixeira, Izabel C. N. de Palmer Paixão, Claudio Cirne-Santos, Dumith Chequer Bou-Habib, Elvira M. Saraiva

**Affiliations:** 1 Departamento de Imunologia, Instituto de Microbiologia Paulo de Góes, Universidade Federal do Rio de Janeiro, Rio de Janeiro, Brazil; 2 Laboratório de Parasitologia Molecular, Instituto de Biofísica Carlos Chagas Filho, Universidade Federal do Rio de Janeiro, Rio de Janeiro, Brazil; 3 Departamento de Biologia Marinha, Instituto de Biologia, Universidade Federal Fluminense, Niterói, Brazil; 4 Departamento de Biologia Celular e Molecular, Instituto de Biologia, Universidade Federal Fluminense, Niterói, Brazil; 5 Laboratório de Imunologia Clínica, Instituto Oswaldo Cruz/FIOCRUZ, Rio de Janeiro, Brazil; 6 Laboratório de Pesquisa sobre o Timo, Instituto Oswaldo Cruz/FIOCRUZ, Rio de Janeiro, Brazil; New York University School of Medicine, United States of America

## Abstract

**Background:**

Chemotherapy for leishmaniasis, a disease caused by *Leishmania* parasites, is expensive and causes side effects. Furthermore, parasite resistance constitutes an increasing problem, and new drugs against this disease are needed. In this study, we examine the effect of the compound 8,10,18-trihydroxy-2,6-dolabelladiene (Dolabelladienetriol), on *Leishmania* growth in macrophages. The ability of this compound to modulate macrophage function is also described.

**Methodology/Principal Findings:**

*Leishmania*-infected macrophages were treated with Dolabelladienetriol, and parasite growth was measured using an infectivity index. Nitric oxide (NO), TNF-α and TGF-β production were assayed in macrophages using specific assays. NF-kB nuclear translocation was analyzed by western blot. Dolabelladienetriol inhibited *Leishmania* in a dose-dependent manner; the IC_50_ was 44 µM. Dolabelladienetriol diminished NO, TNF-α and TGF-β production in uninfected and *Leishmania*-infected macrophages and reduced NF-kB nuclear translocation. Dolabelladienetriol inhibited *Leishmania* infection even when the parasite growth was exacerbated by either IL-10 or TGF-β. In addition, Dolabelladienetriol inhibited *Leishmania* growth in HIV-1-co-infected human macrophages.

**Conclusion:**

Our results indicate that Dolabelladienetriol significantly inhibits *Leishmania* in macrophages even in the presence of factors that exacerbate parasite growth, such as IL-10, TGF-β and HIV-1 co-infection. Our results suggest that Dolabelladienetriol is a promising candidate for future studies regarding treatment of leishmaniasis, associated or not with HIV-1 infection.

## Author Summary

Leishmaniasis is a global disease, which is treated with expensive compounds. Anti-leishmaniasis therapy causes severe side effects, and parasite resistance is often observed, which highlights the importance of discovering new drugs against its causal agent, the protozoan parasite *Leishmania*. Recently, the geographical overlapping distribution of leishmaniasis and the infection by the human immunodeficiency virus type 1 (HIV-1) has contributed to a dramatic increase in the number of individuals infected with *Leishmania*.

The dolabellene diterpene Dolabelladienetriol, purified from the marine algae *Dictyota pfaffii* collected along the northeast coast of Brazil, presents anti-HIV-1 activity. In this paper, we report that Dolabelladienetriol reduces the number of *Leishmania amazonensis* in host cells. We found that Dolabelladienetriol decreased the *Leishmania* load even in the presence of factors that exacerbate parasite growth, such as cytokines and HIV-1 co-infection. Our data may call attention to Dolabelladienetriol as a promising candidate for future studies regarding the treatment of leishmaniasis alone or in association with HIV-1 coinfection.

## Introduction

Leishmaniasis, caused by the *Leishmania* protozoa, comprises a broad spectrum of diseases in humans, characterized by lesions in the skin, mucosa and visceral organs. The form and the severity of the disease are determined by the parasite species and the immunological status of the host [1]. Leishmaniasis affects more than 12 million people in 88 countries in tropical and subtropical areas, causes significant morbidity and mortality and is recognized as one of the most neglected among tropical diseases for which drug development has been encouraged by the Drugs for Neglected Diseases Initiative [2].

Recently, the HIV/AIDS pandemic has contributed to the exponential increase in the incidence of leishmaniasis in endemic areas and, in France and Spain, leishmaniasis is the fourth most prevalent opportunistic disease in the HIV-1-infected populations [3], [4]. These two pathogens infect macrophages and are synergistic; HIV-1 amplifies *Leishmania* multiplication and the severity of leishmaniasis, and *Leishmania* promotes an increase in HIV-1 replication and hastens the progression to AIDS [5], [6]. Importantly, it has been reported that leishmaniasis treatment effectiveness is reduced in *Leishmania*/HIV-1 co-infected patients [3], [4], [7].

*Leishmania amazonensis* is the causative agent of human cutaneous leishmaniasis in the New World. A high proportion of these cases progress to severe anergic diffuse cutaneous leishmaniasis and the visceral form of the disease [8]. Contrary to Europe, mucosal and muco-cutaneous plus cutaneous forms of leishmanisis account for more than 60% of the HIV-*Leishmania* co-infections in Brazil [8].

The first treatment choice for leishmaniasis relies on pentavalent antimonials, and amphotericin B and pentamidine are among the few alternative drugs for resistant cases [1], [7], [9], [10]. All of these drugs have problems that limit their use, such as serious side effects, the induction of parasite resistance, in-patient administration and high costs [9]. The novel drug miltefosine is an effective treatment for visceral leishmaniasis in India. However, miltefosine has shown limited efficacy in other countries and treating other forms of leishmaniasis and is teratogenic [10], [11]. All these findings require the development of new therapeutic agents against leishmaniasis. The picture is similar for the treatment of *Leishmania*/HIV-1 co-infected patients, in whom leishmaniasis clinical relapses are frequent, even in those who receive highly active antiretroviral therapy [3], [4], [7].

Marine organisms constitute a promising source of biologically active compounds [12]. However, few studies have searched for leishmanicidal drugs in algae. Until recently, only ten reports describe the effect of algae extracts against *Leishmania*, but the majority of these studies have used the ability of the extracts to inhibit axenic promastigote or amastigote growth as an endpoint alone. Neither the leishmanicidal activity of the extracts on *Leishmania*-infected macrophages nor their capacity to modulate macrophage functions has been evaluated [13]–[22].

Diterpenes derived from *Dictyota pfaffii*, a brown marine alga of the Dictyotaceae family, are described as having anti-viral, antifungal and antimalarial properties [23]. Moreover, leishmanicidal activity has been demonstrated for *Dictyota* sp. extracts using *L. mexicana* promastigotes and *L. donovani* axenic amastigotes [13], [14]. Recently, dolabellene diterpene 8,10,18-trihydroxy-2,6-dolabelladiene (Dolabelladienetriol) was described as a potent inhibitor of HIV-1 replication in primary human cells *in vitro* [24] by inhibiting the HIV-1 reverse transcriptase enzyme [25].

In this study, we evaluated whether Dolabelladienetriol possess leishmanicidal properties using promastigotes and intramacrophagic amastigotes of *Leishmania amazonensis* as targets of the compound activity. We also analyzed whether Dolabelladienetriol could inhibit parasite survival even in conditions in which its growth is exacerbated, such as in *Leishmania*-infected murine macrophages treated with IL-10 or TGF-β or in human macrophages co-infected by *Leishmania* and HIV-1. We found that Dolabelladienetriol significantly inhibited the multiplication of all *L. amazonensis* forms even when associated with HIV-1 and that Dolabelladienetriol modulated cytokines and nitric oxide production in macrophages.

## Materials and Methods

### Ethics Statement

All of the animal experiments were performed in strict accordance with the Brazilian animal protection law (Lei Arouca number 11.794/08) of the National Council for the Control of Animal Experimentation (CONCEA, Brazil). The protocol was approved by the Committee for Animal Use of the Universidade Federal do Rio de Janeiro (Permit Number: IMPPG 001). The human macrophages were obtained from buffy coats from anonymous normal blood donors and were approved by the Institutional Ethics Committee of the Oswaldo Cruz Foundation (protocol number 397/07).

### Parasites, Virus and Animals

*L. amazonensis* (WHOM/BR/75/Josefa) promastigotes were cultured at 26°C in Schneider insect medium (Sigma) supplemented with 10% fetal calf serum (FCS; Crypion, São Paulo, Brazil) and 40 µg/mL of gentamycin (Schering–Plough, Rio de Janeiro, Brazil).

The monocytotropic, CCR5-dependent isolate HIV-1_Ba-L_ was used for the co-infection assays and was expanded in phytohemagglutinin-activated peripheral blood mononuclear cells as described [5]. BALB/c mice (average weight: 25–30 g) were maintained at 25±5°C on a 12-h day/night cycle and fed a standard pellet diet and water *ad libitum* in our animal facility.

### Dolabelladienetriol

**Figure 1:**
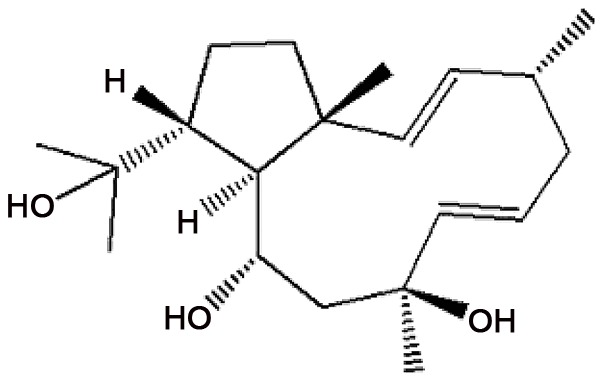
Chemical structure of Dolabelladienetriol (Cirne-Santos et al., 2006).

The compound (1R*,2E,4R*,6E,8S*,10S*,11S*,12R*)-8,10,18-trihydroxy-2,6-dolabelladiene (Dolabelladienetriol) (Figure 1) was obtained by reducing the dolabellane diterpene 10,18-diacetoxy-8-hydroxy-2,6-dolabelladiene as described [23]. The reference specimens of *D. pfaffii* are deposited at the herbarium of the Rio de Janeiro State University (HRJ 9117).

### Dolabelladienetriol effect on *L. amazonensis* promastigotes survival

The promastigotes were incubated in Schneider insect medium supplemented with 10% FCS and 40 µg/mL of gentamycin. The promastigotes were either treated with different Dolabelladienetriol concentrations that were added once to the cultures or untreated. The parasite survival was estimated by counting the viable/motile forms using a hematocytometer during the 5 days of culture at 26°C. The results are expressed as the number of live Dolabelladienetriol–treated parasites, and those maintained in the culture medium and DMSO (Dolabelladienetriol diluents) represented the controls.

### Dolabelladienetriol effect on *L. amazonensis* amastigotes survival

Mouse peritoneal macrophages were stimulated with 3% thioglycolate during 4 days, harvested in RPMI, plated on 13-mm^2^ coverslips inside 24-well plates and allowed to adhere for 2 h at 37°C in 5% CO_2_. The non-adherent cells were removed by washing, and the macrophages were incubated overnight in RPMI supplemented with 10% FCS (Hyclone) at 37°C in 5% CO_2_. The adhered macrophages were then infected with *L. amazonensis* promastigotes (stationary growth phase) at a ratio of 10 parasites:1 macrophage and incubated for 1 h at 35°C in 5% CO_2_. The free parasites were removed by washing with PBS (pH 7.2), and the cultures were maintained for 24 h at 35°C in 5% CO_2_ in RPMI supplemented with 10% FCS. Dolabelladienetriol was added to the cultures and, after a 24 h incubation as described above, the cells were washed, fixed and stained with Giemsa. The number of amastigotes and the percentage of the infected macrophages were determined by counting at least 200 cells in triplicate cultures. The infectivity index was obtained by multiplying the percentage of the infected macrophages by the mean number of amastigotes per infected macrophage as described elsewhere [26].

### Nitric oxide production

Thioglycolate-stimulated mouse peritoneal macrophages were obtained as described above (5×10^5^ cells/well in 24-well plates), or a murine macrophage RAW-264-7 cell line were incubated with 50 µM of Dolabelladienetriol concomitant with 10% IFN-γ (a 4-day culture of the supernatant of an L1210 cell line transfected with the IFN-γ gene) and/or 100 ng/mL of LPS (*E. coli* O111:B4) as described [26]. After 24 h at 37°C in 5% CO_2_, the nitrite concentration in the cell culture supernatants was determined using the Griess method [27]. The reaction was read at 540 nm, and the nitrite concentration determined with reference to a standard curve using sodium nitrite. The results are expressed as micromolar concentrations of nitrite.

### Nitric oxide-trapping capacity

To evaluate the capacity of Dolabelladienetriol to trap nitric oxide, the compound S-nitroso-N-acetyl-D,L-penicillamine (SNAP, Sigma) was used in a cell-free system. SNAP in solution liberates nitric oxide, which is transformed to nitrite [28]. The addition of an NO scavenger to the SNAP solution results in a decay in the supernatant nitrite accumulation. Using this procedure, 50 µM Dolabelladienetriol was incubated with 1 mM SNAP. The positive controls include SNAP and rutin (1 mM, Sigma). After 6-h incubation, media were removed, and the accumulated nitrite was determined using the Griess reaction [27]. The results are expressed as µM of nitrite and compared with the sodium nitrite standard curve.

### Cell viability assays

The murine peritoneal macrophages adhered to 24-well plates and were treated with 100 µM Dolabelladienetriol for 24 h at 37°C in 5% CO_2_. The macrophages were then washed with PBS, incubated with 0.03% Trypan blue solution and scored for viable cells. The effect of Dolabelladienetriol on the mouse peritoneal macrophage, RAW 264-7 cell line and human monocyte-derived macrophage viabilities were determined using the reduction of 2,3-bis[2-methoxy-4-nitro-5-sulfophenyl]-2H-tetrazolium-5-carboxyanilide inner salt (XTT, Sigma) assay as described [29]. Macrophage phagocytosis ability was tested using fluorescein isothiocyanate (FITC)-conjugated zymosan particles (Invitrogen). FITC-zymosan was added to the macrophages, either untreated or treated with either Dollabedienetriol or cytochalasin D (positive control) at a ratio of 4 particles:1 macrophage. The cultures were incubated at 37°C in 5% CO_2_. After 1 h, the monolayers were washed, and the cells were collected in PBS/BSA 1%. To distinguish between the internalized and surface-bound FITC-zymosan, 0.02% Trypan blue in 0.01 M citrate buffer (pH 4.4) was used for quenching the surface-bound fluorescence. The data were collected in a BD FACSCalibur® and analyzed using Summit V4.3 (Dako Colorado, USA). A total of 10,000 gated events were harvested from each sample.

### Cytokine production

The ability of Dolabelladienetriol to induce the production of the cytokines TNF-α and TGF-β was tested using RAW-264-7 cells. These cells were cultured in 24-well plates, either infected with *L. amazonensis* at a ratio of 10 parasites:1 cell or not and incubated for 1 h at 35°C in 5% CO_2_. The free parasites were removed by washing with PBS (pH 7.2), and the cultures were maintained in RPMI supplemented with 5% FCS (Hyclone) for 24 h at 35°C in 5% CO_2_. After a 24-h incubation, the cell monolayers were treated with 50 µM Dolabelladienetriol, and 48 h after treatment, TNF-α and TGF-β production was measured in the cell culture supernatants using a specific sandwich ELISA and capture and detection antibodies obtained from PeproTech (Colonia Portales, Mexico, DF) and Ebioscience (San Diego, CA), respectively, according to the manufacturer's instructions. The assays were performed in duplicate.

### NF-kB Activation

The effect of Dolabelladienetriol on the activation of the transcription factor NF-kB was evaluated by western blot. Accordingly, RAW 264-7 macrophages were either unstimulated or stimulated with LPS (100 ng/ml) and either were untreated or treated with 50 µM Dolabelladienetriol for 1 h. Nuclear extracts were obtained, washed twice with ice-cold PBS and lysed in 50 µL of lysis buffer (50 mM Tris HCl, pH 7.5; 5 mM EDTA; 10 mM EGTA; 50 mM NaF; 20 mM β-glycerophosphate; 250 mM NaCl; 0.1% Triton X-100; 1 µg/mL BSA and 1∶500 of protease inhibitor cocktail II [Calbiochem]) for isolating the total protein. The total nuclear proteins (10 µg) were subjected to electrophoresis in 10% SDS-polyacrylamide gels and electrophoretically transferred to a nitrocellulose membrane (Amersham). After blocking with 5% non-fat dry milk in TBS containing 0.15% Tween-20 (TBS-T), the blots were incubated with 1 µg/mL anti-p65 antibody (SC372, Santa Cruz), followed by horseradish peroxidase-anti-rabbit IgG (1∶3000, Sigma) treatment and detected using an ECL chemiluminescent detection system (Amersham).

### Macrophage infection with HIV-1 and *Leishmania*

These assays were performed using human monocyte-derived macrophages (MDMs), which can be infected by *Leishmania* and HIV-1 [5]. Initially, MDMs were obtained from peripheral blood mononuclear cells (PBMCs), which were isolated using density gradient centrifugation (Histopaque, Sigma) from buffy coat preparations of healthy blood donors as described [5]. Briefly, 1×10^6^ PBMCs were seeded in Permanox chamber slides (8 wells; Nalge Nunc) in DMEM with 10% human serum (Sigma). The cells were cultured for 6–8 days for monocyte differentiation in the macrophages. The non-adherent cells were removed, and the remaining macrophages were nourished with DMEM as described [5]. The macrophage purity was greater than 90% as determined using flow cytometry analysis (FACSCalibur flow cytometer, Becton Dickinson, San Jose, CA) using anti-CD3 (Caltag Laboratories Inc., Burlingame, CA) and anti-CD16 (Southern Biotech, Birmingham, AL) monoclonal antibodies. The macrophages were infected with the HIV-1 R5 isolate Ba-L using 5 to 10 ng/mL HIV-1 p24 antigen. After an overnight incubation, the excess virus was removed by washing, and fresh medium was added; the infected macrophages were maintained under standard culture conditions. After 7 to 8 days of HIV-1 infection, the macrophages were infected with *L. amazonensis* stationary phase promastigotes using a ratio of 3 parasites:1 cell at 34°C for 16 h. The non-internalized promastigotes were removed by washing, and fresh medium was added. The cultures were treated either with Dolabelladienetriol or amphotericin B or were untreated and maintained at 37°C in 5% CO_2_ for 4 to 5 days. The supernatants were collected, and to measure HIV-1 production, the HIV-1 p24 antigen levels were assessed using a commercial ELISA kit (ZeptoMetrix Co., Buffalo, NY). The slides were then fixed with methanol and stained with Giemsa. To evaluate *Leishmania* replication, amastigote forms of *Leishmania* inside the infected-macrophages were counted using light microscopy. The results are expressed as infectivity index, which was calculated by multiplying the percentage of infected macrophages by the average number of parasites per macrophage. Because the infectivity index values varied between experiments, these values were normalized.

### Statistical analysis

The data were analyzed using either the Student's t-test when comparing two groups or a one-way ANOVA for more than two groups using the GraphPad Program. *p* values of less than 0.05 were considered significant.

## Results

**Figure 2:**
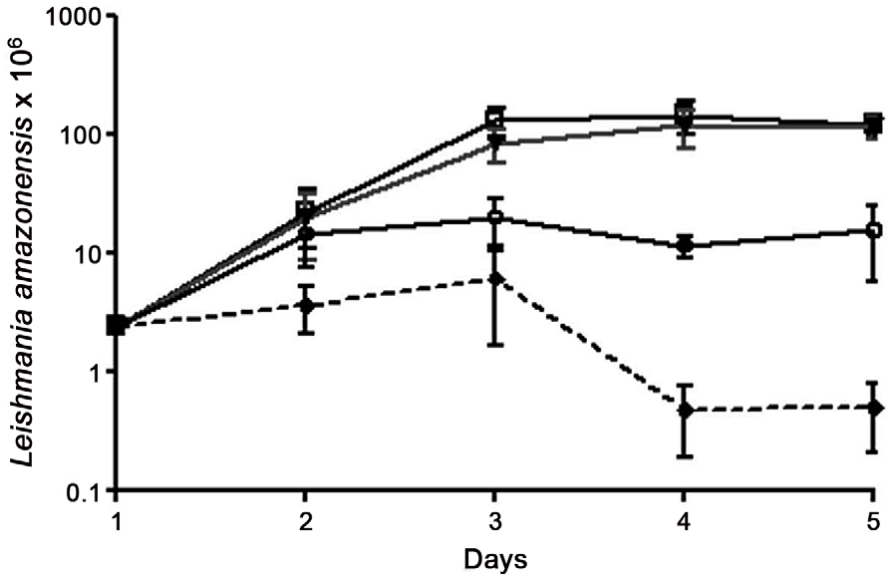
Leishmanicidal activity of Dolabelladienetriol against *Leishmania amazonensis* promastigotes. Promastigote cultures were treated with Dolabelladienetriol, and parasite viability was counted daily. Dolabelladienetriol 50 µM (open circle) and 100 µM (black diamond); control (open square) and DMSO vehicle (black triangle). The data represent the mean+SEM of three independent experiments. *p*<0.05 for the control vs. Dolabelladienetriol 50 µM and 100 µM and for Dolabelladienetriol 50 µM vs. Dolabelladienetriol 100 µM.

**Figure 3:**
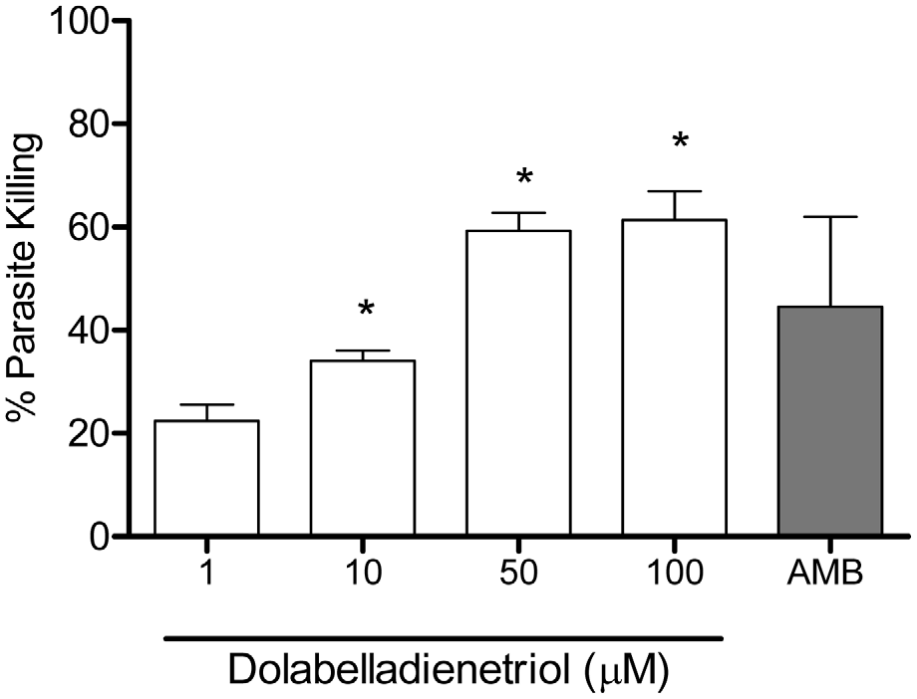
Leishmanicidal activity of Dolabelladienetriol on intracellular amastigotes. *L. amazonensis*-infected mouse peritoneal macrophages were treated with the indicated concentrations of Dolabelladienetriol (open bars) or 1.1 µM amphotericin B (grey bar). Amastigote growth was assessed 24 h after Dolabelladienetriol treatment. The results from three experiments in duplicates are shown as a percentage of parasite death+SEM compared with the untreated control. * *p*<0.05.

Initially, we investigated the anti-*Leishmania* activity of Dolabelladienetriol on stationary-phase promastigotes of *L. amazonensis* exposed to this drug by counting viable parasites. Our results show that concentrations of 50 µM and 100 µM of Dolabelladienetriol, added once to the cultures, inhibited 84% and 95.5% of promastigote growth, respectively, at 3 days after the treatment (Figure 2). Based on the efficacy of Dolabelladienetriol against *Leishmania* promastigotes, we then evaluated the effectiveness of Dolabelladienetriol against amastigote intracellular forms. Therefore, peritoneal macrophages were infected with *L. amazonensis* for 24 h and then treated with different concentrations of Dolabelladienetriol. The parasite growth was measured at 24 h after the treatment. We found that Dolabelladienetriol repressed the amastigote multiplication in a dose-dependent manner, inhibiting 56% and 61% of growth at concentrations of 50 µM and 100 µM of Dolabelladienetriol, respectively. The IC_50_ of Dolabelladienetriol was 43.9 µM. Amphotericin B (1.1 µM) was used as a control and inhibited 44.5% of the amastigote survival (Figure 3). Altogether, these results demonstrate the potent leishmanicidal activity of Dolabelladienetriol, regardless of the parasite form.

**Figure 4:**
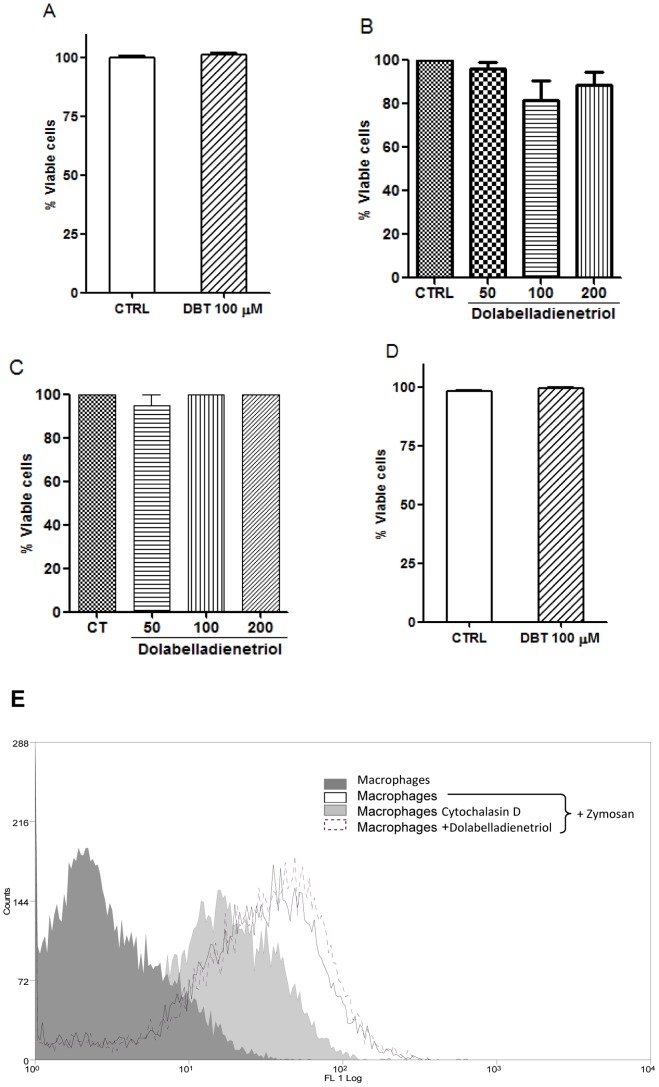
Dolabelladienetriol safety for macrophages. Adhered mouse peritoneal macrophages (**A, D**), RAW 264.7 cells (**B, E**) and human monocyte-derived macrophages (**C**) were either treated with Dolabelladienetriol at the indicated concentrations or untreated (CTRL), and the cell viability was measured using the XTT reduction assay (**A, B, C**), the Trypan blue exclusion test (**D**), and phagocytosis of FITC-labeled zymosan (**E**). The results from two independent experiments performed in duplicate are expressed as a percentage of the viable cells+SEM (**A–D**). One representative experiment of zymosan phagocytosis out of two experiments performed in duplicate is shown (**E**). *p<0.05.

In parallel with the anti-leishmanial studies, we evaluated the safety of administering Dolabelladienetriol to host cells using the XTT method. Mouse peritoneal macrophages, RAW 264.7 cells and human monocyte-derived macrophages were used to verify whether this drug would induce mitochondrial cell damage (Figures. 4A, 4B, 4C). Our results indicate that Dolabelladienetriol cannot affect the mitochondrial activity of all of the cells tested. In addition, Dolabelladienetriol did not affect the membrane integrity of mouse peritoneal macrophages, as tested using the Trypan blue exclusion assay (Figure 4D). Moreover, 100 µM of Dolabelladienetriol did not affect phagocytosis of the FITC-labeled zymosan particles in the RAW 264.7 macrophages (Figure 4E). Cytochalasin D, an inhibitor of actin polymerization, significantly decreased the zymosan phagocytosis of the macrophages, as expected (Figure 4E).

**Figure 5:**
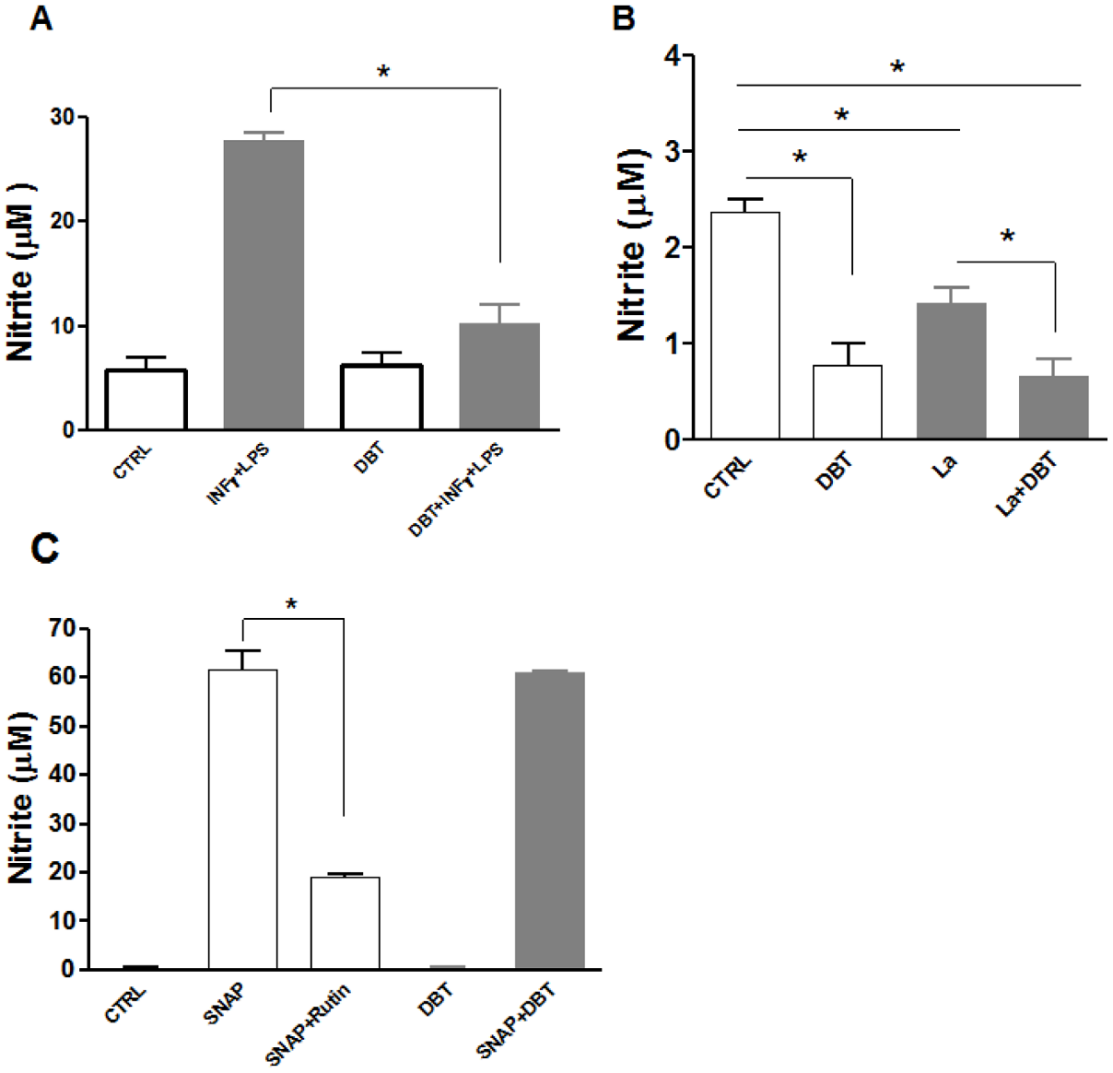
Effect of Dolabelladienetriol on nitric oxide (NO) production. (**A**) Non-activated mouse peritoneal macrophages (open bars) and IFN-γ and LPS-activated macrophages (grey bars) were treated with 50 µM Dolabelladienetriol (DBT), which was added simultaneously with IFN-γ and LPS. (**B**) *L. amazonensis* (La) infected (grey bars) or uninfected (open bars) RAW 264.7 cells were treated or not with 50 µM Dolabelladienetriol 24 h after infection. NO production in **A** and **B** was measured in the supernatants that were harvested 48 h after treatment using the Griess assay. (**C**) Dolabelladienetriol scavenger activity. A cell-free system using SNAP as an NO donor was either incubated with 50 µM of Dolabelladienetriol (DBT) or untreated. Rutin (1 mM), an NO scavenger, was used as a positive control. The nitrite concentration was determined using the Griess reaction. The data represent the mean+SEM of two different experiments performed in triplicate. *p<0.05.

Considering that nitric oxide (NO) is a potent leishmanicidal agent, we investigated whether Dolabelladienetriol could modulate this mechanism in macrophages. Our results demonstrated that Dolabelladienetriol could not stimulate NO production in thioglicolate-elicited peritoneal macrophages and inhibited 63% of the NO production when the macrophages were stimulated with IFN-γ and LPS (Figure 5A). Dolabelladienetriol also inhibited 67% of the basal NO production of the RAW macrophages (Figure 5B). To further investigate the ability of Dolabelladienetriol to inhibit NO production, the NO level was measured in the supernatants of the *Leishmania*-infected RAW cells that were untreated and Dolabelladienetriol-treated. A 50% reduction in NO production was observed in the *Leishmania*-infected RAW cells that were treated with 50 µM Dolabelladienetriol. The untreated and Dolabelladienetriol-treated cell-free systems were used with S-nitroso N-acetyl-D,L-penicillamine (SNAP) as the NO donor to eliminate the NO scavenger effect. The addition of rutin, an NO trapping substance, to the SNAP solution reduced the NO levels by 69%, whereas the addition of 50 µM Dolabelladienetriol to SNAP did not reduce the NO levels (Figure 5C), indicating that Dolabelladienetriol was not an NO scavenger. Therefore, NO might not participate in the Dolabelladienetriol-mediated leishmanicidal effect.

**Figure 6:**
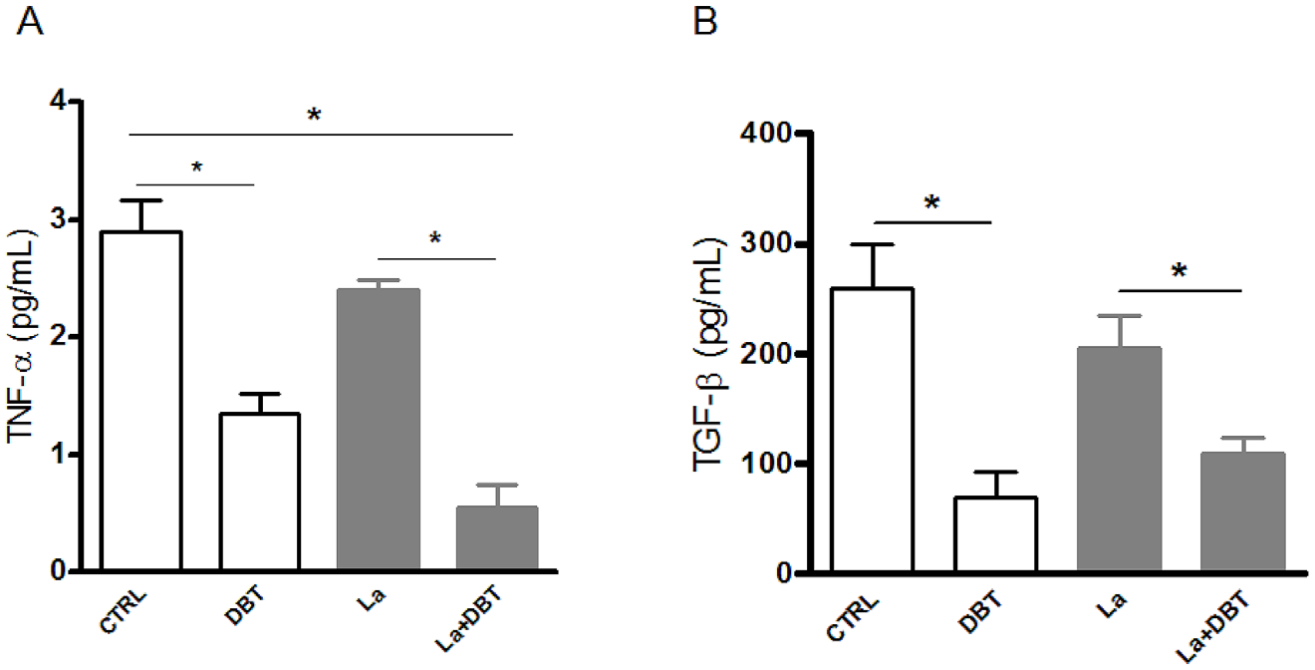
Cytokine production inhibition by Dolabelladienetriol. *L. amazonensis* (La) **i**nfected or non-infected RAW 264.7 cells were treated or not with 50 µM of Dolabelladienetriol (DBT) 24 h after infection. (**A**) TNF-α and (**B**) TGF-β production was determined using ELISA assays 48 h after DBT treatment. The data represent the mean of two experiments in triplicate+SEM. * *p*<0.05.

It has been reported that macrophage-produced cytokines can affect the intracellular growth of *Leishmania*. For example, TNF-α inhibits parasite multiplication [30], whereas TGF-β increases it [31]. Therefore, to evaluate whether Dolabelladienetriol could modulate the macrophage production of these cytokines, the TNF-α and TGF-β levels were measured in supernatants from uninfected or infected RAW macrophages, untreated or treated with 50 µM Dolabelladienetriol. TNF-α production was inhibited by 54% in the uninfected cells and 77% in the *Leishmania*-infected RAW cells that were Dolabelladienetriol-treated (Figure 6A). TGF-β production decreased by 73% in the uninfected Dolabelladienetriol-treated cells. In parallel, we observed that the amount of TGF-β present in the RAW cells cultured with 5% FCS was 4.5-fold higher than the amount present in the cell-free culture medium with 5% FCS. This result demonstrates that Dolabelladienetriol inhibits *de novo* TGF-β production (data not shown). Similarly, in the *Leishmania*-infected RAW macrophages, TGF-β levels declined by 47% in the Dolabelladienetriol-treated samples compared with the untreated controls and by 58% compared with the untreated and uninfected controls (Figure 6B). Although, Dolabelladienetriol treatment did not induce an increased production of the anti-*Leishmania* cytokine TNF-α, the inhibition of TGF-β a *Leishmania*-enhancing cytokine, could partially explain the effect of the treatment.

**Figure 7:**
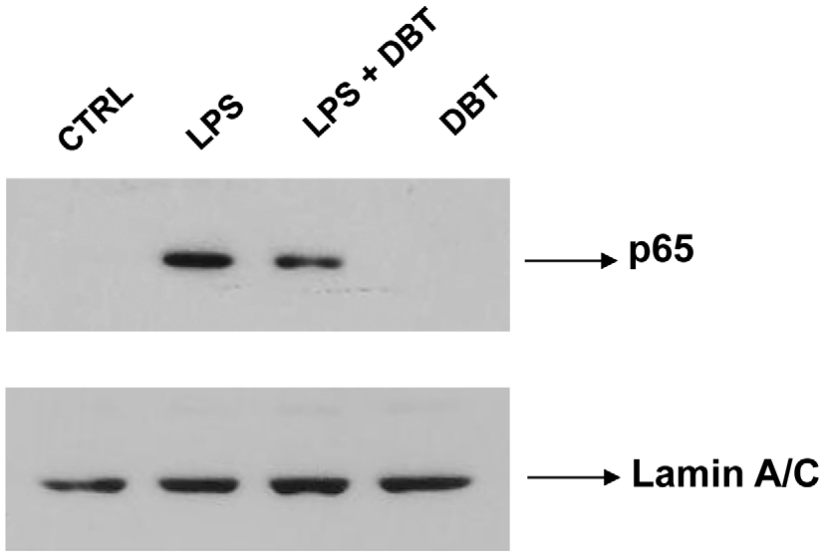
Dolabelladienetriol inhibits NFκB nuclear translocation. RAW264.7 macrophages were left untreated (CTRL) or stimulated with LPS, and additionally treated or not with 50 µM Dolabelladienetriol (DBT) for 1 h. The total nuclear proteins were detected by blotting using p65 and lamina A/C specific antibodies.

The transcription factor NF-kB is involved in conditions that favor *Leishmania* growth [32]. Therefore, it is of interest to test if Dolabelladienetriol prevents NF-kB activation. Therefore, nuclear extracts from the RAW macrophages that were either non-stimulated or stimulated with LPS and either untreated or treated with Dolabelladienetriol were used to determine by blotting if the NF-κB p65 subunit would translocate to the nucleus. The results depicted in Figure 7 indicate that Dolabelladienetriol reduced the nuclear translocation of NF-κB in macrophages stimulated by LPS, a classical NF-kB activator.

**Figure 8:**
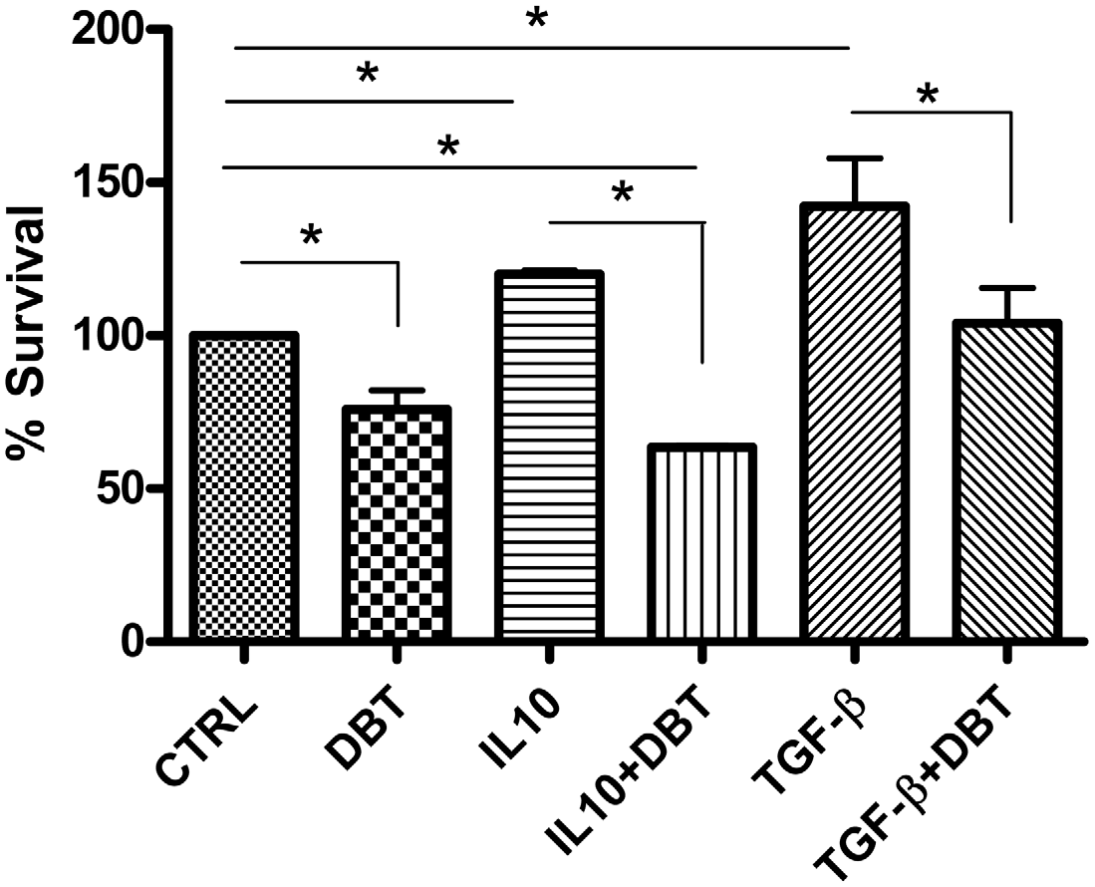
Leishmanicidal activity of Dolabelladienetriol in conditions of parasite exacerbated growth. *L. amazonensis* infected mouse peritoneal macrophages were treated with IL-10 (10 ng/ml) or TGF-β (5 ng/ml), and additionally treated or not with 50 µM Dolabelladienetriol (DBT). The parasite growth was assessed at 48 h after the DBT treatment. The results from two independent experiments in duplicate for IL-10 and four experiments in duplicate for TGF-β are shown as the percentage of parasite survival+SEM compared with the untreated control. * *p*<0.05.

*Leishmania* replication in macrophages is reported to be stimulated by TGF-β and/or IL-10 [31]. Therefore, whether Dolabelladienetriol could inhibit parasite growth stimulated by either cytokine was analyzed. Infected macrophages were treated with each cytokine and subsequently treated with Dolabelladienetriol to test this hypothesis. The results depicted in Figure 8 show that Dolabelladienetriol decreased *Leishmania* replication even when parasite growth was exacerbated by either IL-10 or TGF-β. In our studies, adding either IL-10 (10 ng/mL) or TGF-β (5 ng/mL) to infected macrophages increased parasite survival by 20% and 42%, respectively, compared with the non-stimulated control. In contrast, when the infected macrophages were stimulated with either IL-10 or TGF-β and were treated with 50 µM Dolabelladienetriol, the parasite survival decreased by 57% and 38%, respectively, compared with the infected Dolabelladienetriol-untreated macrophages that were stimulated with either IL-10 or TGF-β. The killing observed after Dolabelladienetriol treatment in these assays (29%) was smaller than that observed in the experiments depicted in the figure 3 (56%). This difference on *Leishmania* survival can be explained by the fact that the parasites that survived the exposure to Dolabelladienetriol during the first 24 h (results shown in Figure 3) continued to multiply in infected cells, thus resulting in a smaller percentage of killings when the survival was measured after 48 h of Dolabelladienetriol treatment (Figure 8).

**Figure 9:**
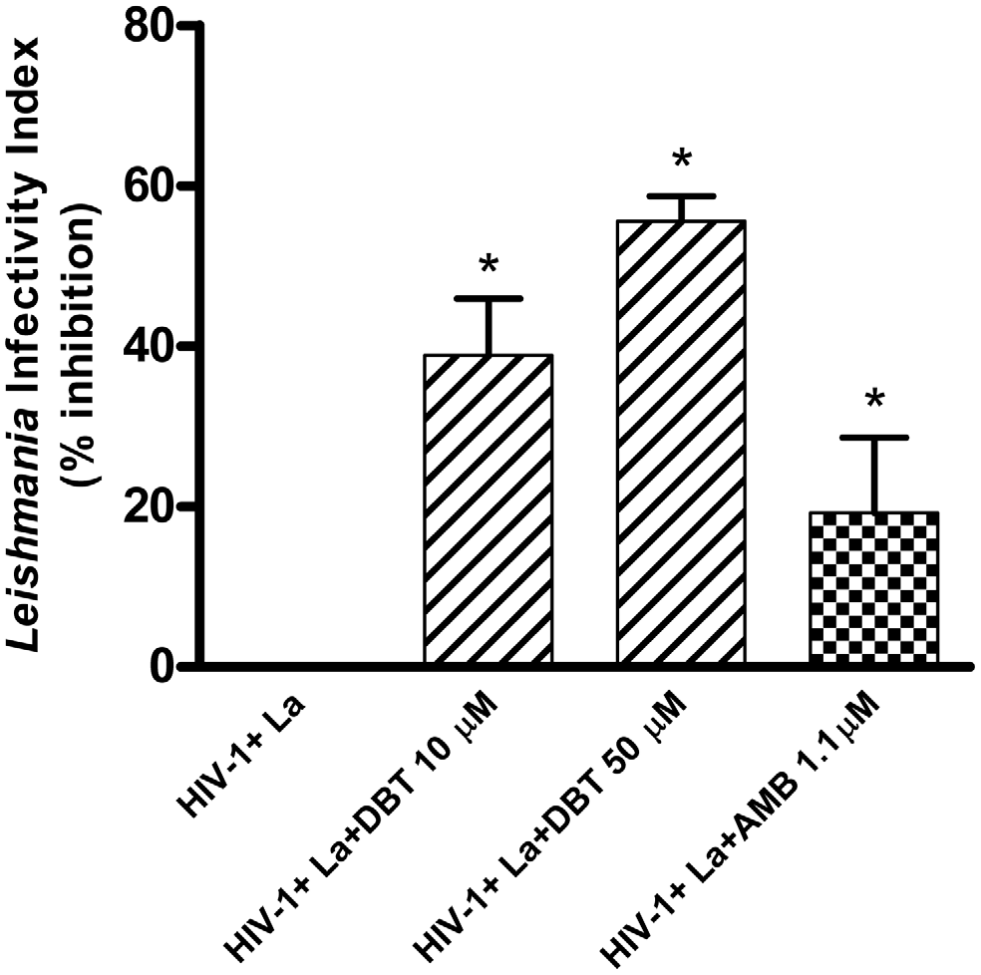
Leishmanicidal effect of Dolabelladienetriol in the *Leishmania*/HIV-1 co-infected macrophages. Human macrophages were infected with HIV-1 for 7–9 days, followed by *Leishmania* (La) infection (a ratio of 3 parasites:1 cell). After 72 h, the macrophages were either treated with 10 µM or 50 µM Dolabelladienetriol (DBT) or 1.1 µM amphotericin B (AMB) or untreated. The infectivity index of *Leishmania* was determined by randomly counting at least 200 cells in each condition. The data represent the means+SEM of four different donors in the experiments performed in duplicate. * *p*<0.05 compared with *Leishmania* growth in the untreated co-infected macrophages. Viral production in the *Leishmania*-HIV-1 co-infected macrophages: 48.96±16.05 ng/m.

We previously reported that Dolabelladienetriol inhibits HIV-1 replication [24], [25] in human primary macrophages. Therefore, we analyzed whether Dolabelladienetriol could inhibit *Leishmania* growth in macrophages that were simultaneously co-infected by HIV-1, a condition that induces exacerbated viral and protozoan growth, as previously described [5]. Consequently, primary human macrophages were infected by both pathogens and treated with Dolabelladienetriol. Parasite load and virus replication were assessed by determining the *Leishmania* infectivity index and measuring the HIV-1 p24 antigen in the culture supernatant, respectively. The parasite growth in *Leishmania*-HIV-1 co-infected macrophages was reduced by 39% and 56% after Dolabelladienetriol treatment with 10 µM and 50 µM, respectively (Figure 9). Amphotericin B, used for comparison, was much less potent in this system and reduced the parasite load by 19% in the co-infected cells (Figure 9). In agreement with our previous reports [5], HIV-1 doubled *Leishmania* growth. Additionally, Dolabelladienetriol potently reduced the HIV-1 replication in the co-infected macrophages, reaching up to 99% inhibition with a 50 µM treatment (data not shown), similarly to what we described elsewhere [24], [25].

## Discussion

In the present study, we have shown for the first time that a compound purified from *Dictyota pfaffii* algae possesses leishmanicidal activity. Dolabelladienetriol, a dolabellane diterpene, was active against *Leishmania amazonensis* in an isolated infection and during HIV-1 co-infection in primary murine and human macrophages, respectively.

Initially, we demonstrated that Dolabelladienetriol was active against the parasite, significantly inhibiting promastigotes growth. This result suggested that Dolabelladienetriol could directly kill the parasite. Importantly, Dolabelladienetriol was active against intramacrophagic amastigotes, the forms that maintain the infection in mammalian hosts. Dolabelladienetriol anti-amastigote activity was dose-dependent and lacked host cell toxicity, as measured using three different methods. Dolabelladienetriol did not cause toxicity in the murine macrophages even when tested at a 1 mM concentration using the XTT assay (data not shown). No toxicity was found in the human monocytes derived-macrophages until a 200 µM dose of Dolabelladienetriol, as measured using the XTT assay. As previously reported, no toxicity was found in the human monocyte-derived macrophages until a 500 µM dose of Dolabelladienetriol, as measured using the Trypan blue exclusion test [24].

Of the few reports that have analyzed algae anti-*Leishmania* activity, most of these studies have characterized the leishmanicidal effect of algae extracts in axenic promastigotes and amastigotes [13]–[17], [19], [21]. The anti-leishmanial activity of a pure, isolated drug from algae has been reported for elatol and obtusol, obtained from the red seaweed *Laurencia dendroidea* [18], [21].

Dolabelladienetriol is included in the large family of terpenes, which contains other members possessing antileishmanial properties, including sesquiterpenes, diterpenoids and triterpenes. These terpenes can affect the actions of *Leishmania*, for example, in the cell cycle, protease activity, lipid synthesis or indirectly by modulating macrophage activation [33]–[37]. Although the mechanism(s) responsible for the anti-leishmanial effect of Dolabelladienetriol is/are unknown, we demonstrate that this drug modulates macrophage activity by inhibiting NO, TGF-β and TNF-α production. TNF-α and NO are important mediators for *Leishmania* killing in macrophages [38]. Dolabelladienetriol treatment decreased TNF-α and NO production, both in infected and uninfected macrophages. TNF- α is an important cytokine that induces nitric oxide synthase expression, promotes NO production and parasite death [39]; however, evidence of persistent leishmaniasis caused by a strong inflammatory response induced by high TNF-α levels were reported [39]–[42]. Moreover, the clinical improvement of leishmaniasis patients treated with pentoxifylline, a TNF-α inhibitor, has been shown [43]–[45]. Allenbach et al. [45] demonstrated self-healing leishmaniasis in soluble TNF-α knockout (KO) mice. However, the same phenotype was not observed in soluble and membrane TNF-α knockout mice; these mice could not cure their infection. These findings suggest that membrane TNF-α plays an important role in leishmaniasis resolution. Similar to pentoxifylline, Dolabelladienetriol could be an important tool for leishmaniasis control that occurs with high TNF-α levels through its capacity to inhibit this cytokine. Because TNF-α gene expression is NF-kB controlled, NF-kB modulation by Dolabelladienetriol was investigated. Our results demonstrated that Dolabelladienetriol could inhibit NF-κB activation in RAW 246-7 macrophages that were either unstimulated or stimulated with LPS. Marine natural products have been recognized as a promising source of NF-κB inhibitors; among the algae products tested, 75% could inhibit NF-kB [46]. It has been demonstrated that natural sea products can inhibit NF-κB activation by different mechanisms, such as the modulation of the NF-κB inhibitor (IκB) or the IκB kinase (IKK), NF-kB ubiquitination and 26S proteasome degradation [47]. Although the mechanism is unknown, Dolabelladienetriol-mediated NF-κB inhibition includes this drug in a group of molecules with anti-inflammatory properties.

Recently, we demonstrated that an indole alkaloid-enriched fraction from *Tabernaemontana catharinensis* inhibited TGF-βproduction and possessed anti-*Leishmania amazonensis* activity [26]. Similarly, we detected that Dolabelladienetriol inhibited TGF-β production in normal or *Leishmania*-infected macrophages in association with anti-leishmanial activity. The favorable role of TGF-β on the development of *Leishmania* infection is well established in the literature. For example, the administration of exogenous TGF-β to *Leishmania*-infected mice leads to lesion enhancement, whereas treatment with anti-TGF-β antibody has a protective effect [48]. High TGF-β levels are involved in anthroponotic cutaneous leishmaniasis, which is caused by *L. tropica*, and post-kala-azar dermal leishmaniasis, which is caused by *L. donovani* [31], [49]. Additionally, the neutralization of TGF-β reverses disease susceptibility in murine leishmaniasis, even in the presence of high IL-10 levels [50], [51]. Importantly, our results show that Dolabelladienetriol was effective even when parasite growth was exacerbated by TGF-β and IL-10.

Dolabelladienetriol was also active against *Leishmania* in HIV-1 co-infected human macrophages. We previously described that an HIV-1 infection exacerbates the *Leishmania* load in human macrophages, an effect that is mediated by the HIV-1 transcriptional transactivator (Tat) protein through TGF-β production [5]. This effect was reversed by adding anti-TGF-β to the infected macrophage cultures [5]. Thus, our present results indicate that Dolabelladienetriol-mediated inhibition of TGF-β production plays an important role in the activity of this compound against *Leishmania* in the *Leishmania*/HIV-1 co-infected macrophages.

Overall, the leishmanicidal and anti-HIV-1 activities possessed by Dolabelladienetriol support this compound as a promising candidate for future studies regarding the treatment of *Leishmania* infection, either in isolated episodes or in association with HIV-1, a condition in which growth of both pathogens is exacerbated. Because *Leishmania*-HIV-1 co-infection is an emerging health problem in several countries, drugs that can inhibit both pathogens should be developed.
